# Spontaneous cecal perforation in a 40-year-old pregnant woman treated by primary repair and omental patch: a case report

**DOI:** 10.1186/s13256-017-1336-x

**Published:** 2017-06-18

**Authors:** Cyrille Kouam, Ouasso Passang, Marc-Leroy Guifo, Nkolaka Atem

**Affiliations:** 1 Faculty of Medicine and Biomedical Sciences, University of Yaoundé I, Yaoundé, Cameroon; 2 Regional Hospital of Bafoussam, Bafoussam, Cameroon; 3 University Teaching Hospital, Yaoundé, Cameroon

**Keywords:** Cecum, Perforation, Primary suture, Right hemicolectomy

## Abstract

**Background:**

Spontaneous colonic perforations are scarce, and cecal perforations even more so. Preoperative diagnosis of the latter in a pregnant woman is particularly difficult because of physiologic changes and restrictions on some diagnostic imaging techniques, such as X-rays. Furthermore, management of these patients is a big challenge.

**Case presentation:**

We present a case of a spontaneous cecal perforation in a 40-year-old pregnant black woman in the Regional Hospital of Bafoussam in Cameroon. The results of clinical examination and ultrasonography on admission were in line with acute generalized peritonitis in a woman at 20 weeks of a viable pregnancy, indicating an urgent laparotomy. Operative findings were a 1 × 1-cm perforation on a distended cecum with minimal fecal contamination. The treatment consisted of excision of the edges, primary suture of the perforation, and omentoplasty. The recovery of the patient was uneventful.

**Conclusions:**

The management of spontaneous cecal perforation in a pregnant woman was a big challenge. The perforation was repaired by primary suture and omentoplasty. Further studies comparing this approach with right hemicolectomy are recommended.

## Background

Spontaneous perforation of the colon is defined as a sudden perforation of an apparently healthy colon in the absence of any other disease or injury. Fewer than 100 cases have been reported in the literature to date [1]. More than 60% of these perforations occur in the sigmoid colon, making spontaneous cecal perforation a very uncommon condition [1, 2]. The latter is difficult to assess prior to surgery and is even more difficult in a pregnant patient, owing to the peculiar anatomic and physiologic features of pregnancy and the restrictions imposed on diagnostic imaging techniques such as radiography and computed tomography (CT) [3]. The management of a cecal perforation in a pregnant patient is a great challenge, especially regarding the choice of surgical technique (primary repair + omental patch versus right hemicolectomy) [4]. In this report, we present a case of spontaneous cecal perforation in a 40-year-old pregnant woman treated by primary repair and omental patch in the Regional Hospital of Bafoussam in Cameroon.

## Case presentation

Our patient was a 40-year-old multiparous pregnant black woman at a gestational age of 20 weeks who was not compliant with antenatal care. She had no history of weight loss, chronic constipation, or blood per rectum, nor did she or her relatives report a previous laparotomy. She was brought to our institution’s emergency department by her family 6 days after the onset of a progressively painful abdominal distention, bilious vomiting with fever, and asthenia. She had not passed stools for 3 days prior to her admission. She had no urinary symptoms, and she reported feeling active fetal movements.

On physical examination, the patient was conscious and had the following parameters: blood pressure 109/55 mmHg, pulse 125 beats/minute, respiratory rate 25 breaths/minute, and body temperature 37.7 °C. She presented with abdominal distention with no scars or skin lesions. She had a generalized guarding with rebound tenderness. The evaluation by the obstetrician revealed a uterine height of 22 cm. The patient’s umbilical and inguinal orifices were not patent. Her bowel sounds were markedly diminished. On digital rectal examination, her pouch of Douglas was extremely tender, and the gloves were soiled with normal feces. Elsewhere, the patient’s physical examination was unremarkable.

The diagnosis of acute generalized peritonitis in a 20-week gestational age pregnancy was retained with an indication of urgent laparotomy. Abdominal ultrasonography revealed a viable, 21-week gestational age intrauterine monofetal pregnancy with no etiologic finding that could explain the occurrence of peritonitis. Neither plain abdominal x-ray nor abdominal CT was performed. The pretherapy workup revealed hyperleukocytosis at 18,000/mm^3^ with neutrophilia and a hemoglobin level of 12 g/dl. The patient’s electrolyte profile was normal, with a K^+^ level of 3.9 mEq/L. Her coagulation profile was normal, and the result of her human immunodeficiency virus (HIV) serology was negative. She was classified as American Society of Anesthesiologists Physical Status classification II and Altemeier wound classification 4 after preanesthetic consultation.

**Fig. 1:**
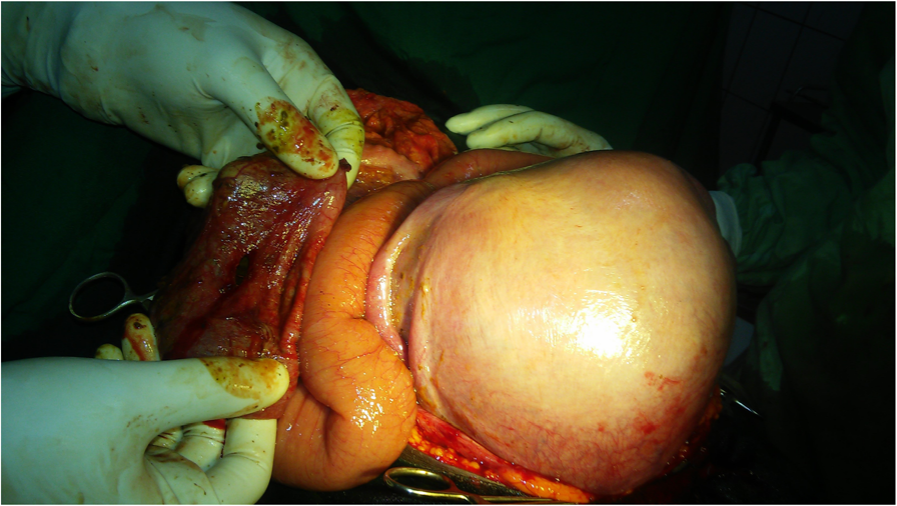
A view of the cecal perforation after excision of the edges

The treatment was aimed at suppressing the cause of the peritonitis, decreasing the peritoneal bacterial contamination to its minimum, and addressing the complications of the disease if present. Preoperative resuscitation was initiated with the use of crystalloids. A nasogastric tube and urinary catheter were inserted. We performed an exploratory laparotomy. Perioperative findings included a more or less macroscopically normal peritoneal cavity and a 1 × 1-cm perforation on the anterolateral aspect of a slightly inflamed and distended cecum (Fig. 1). No obstruction of the distal colon was noted. The patient’s appendix was normal. The perforation was repaired after excision of the edges by primary suture and omental patching. An appendectomy followed by an abundant abdominal toilet with normal saline was performed. The abdominal cavity was drained with two tubes inserted into the right paracolic gutter and pouch of Douglas. The patient received perioperative venous thromboembolic disease prophylaxis, analgesics, and antibiotics targeting Enterobacteriaceae and gram-negative bacteria. The postoperative period was uneventful, with early ambulation on day 1 and onset of enteral feeding on day 3. The results of the patient’s postoperative obstetric evaluation were normal. The drains were removed on day 6. The patient was discharged on day 10 and referred to an obstetrician for the continuation of antenatal care.

## Discussion

Acute abdomen in pregnancy is a quite challenging diagnostic and therapeutic issue because it raises medical and ethical concerns [5]. Common etiologies of secondary peritonitis in sub-Saharan countries include appendicitis, peptic ulcer perforation, and typhoid fever. Colonic perforations resulting in severe peritonitis are uncommon and could be the result of appendicitis, diverticulitis, neoplasms, or acute pseudo-obstruction (Ogilvie syndrome) [4]. Spontaneous perforations of the colon are scarce and are defined as sudden perforation of an apparently healthy colon in the absence of any other disease or injury [1]. Spontaneous cecal perforations are even more uncommon [6]. The diagnosis of acute abdomen, and a spontaneous cecal perforation even more so, in a pregnant patient can be particularly difficult because of physiologic changes that occur in pregnancy and owing to restrictions on some imaging techniques, such as X-rays and CT [7]. Thus, the management of a condition that is straightforward in the nonpregnant state can become very complicated. Our report of this case of spontaneous cecal perforation in a 40-year-old multiparous pregnant woman draws attention to a rare clinical entity raising diagnostic and therapeutic challenges.

The ileocecal region is one of the vulnerable anatomic points of the colonic vasculature as well as the splenic flexure (Griffiths point) and the rectosigmoid region (Sudeck point) [1]. The cecum is more susceptible to perforation, owing to its thin wall and its large diameter, allowing it to expand three times more than other areas of the colon. According to the law of Laplace, the intraluminal pressure needed to stretch the wall of a hollow tube is inversely proportional to its radius. This tension causes stretching of the cecal vessels, followed by occlusion and ischemia, which could result in necrosis. Cecal dilation depends on a competent ileocecal valve to prevent retrograde decompression of the cecum and proximal colon. The cecum is at risk of perforation if dilated to more than 12 cm [8]. In 1984, Berry *et al*. classified spontaneous perforations of the colon into stercoral and idiopathic types [6]. The stercoral perforation, commonly seen in chronic constipation, is described as a round or ovoid hole with necrotic and inflammatory edges associated with an ulcerative lesion [1, 6]. The idiopathic type, described as a linear tear with a normal appearance of the colonic wall, is less common and has a better prognosis owing to minimal fecal contamination [1, 6]. A vascular theory suggesting a combination of hypoperfusion of colonic tissue and constitutional weakness of the bowel wall has been proposed to explain idiopathic perforations [1]. Spontaneous colonic perforations can be associated with some conditions, such as chronic constipation, fecal impaction, or bowel hypomotility, encountered during pregnancy [1].

Preoperative diagnosis of cecal perforation is infrequent, and it is more often an operative finding, as observed in our patient. The assessment of acute surgical conditions is often made more difficult during pregnancy, owing to the deviation of organs caused by an enlarged uterus and relaxation of the abdominal wall [3]. The laxity of the abdominal muscles that commonly occurs in the later part of pregnancy often makes it difficult to appreciate the physical findings of abdominal rigidity, guarding, and rebound tenderness. Gastrointestinal motility disorders in pregnancy are caused primarily by hormonal changes and not by the physical effects of the gravid uterus [9]. Pregnancy slows the intestinal transit, and pseudo-obstruction is particularly frequent [10]. These effects are mediated by progesterone, with estrogen probably acting as a primer [9].

Neither plain abdominal x-ray nor abdominal CT were performed for our patient, owing to restrictions on these imaging techniques during pregnancy. Abdominal X-rays are controversial regarding their effect on the fetus [3]. During pregnancy, other imaging procedures not associated with ionizing radiation (e.g., ultrasonography and magnetic resonance imaging) should be considered instead of radiography whenever possible. Ultrasonography is the first choice in making a diagnosis of acute abdomen in pregnancy because it is affordable and noninvasive to both mother and fetus, and also because a lot of information can be obtained by this simple procedure [3]. Thus, abdominal examination remains of paramount importance in the diagnostic process of acute abdomen in pregnancy.

A big question to answer in such a case is the choice of surgical technique for repairing the cecal perforation. In our patient, we found a small hole (1 × 1 cm) and minimal fecal contamination of the peritoneal cavity. After the edges were excised, the perforation was repaired by using a primary suture and omental patch. The surgical approach in treating a cecal perforation varies, depending on the primary etiology: primary suture, cecostomy, or right hemicolectomy [2]. Only a few reports in the literature compare the two main approaches: right hemicolectomy versus suture and omental patch [4]. Less invasive management (suture repair + omental patch) with postoperative antibiotics is recommended for uncomplicated perforation, absence of severe infection, and well-controlled local hemostasis [4]. In cases of severe inflammation, torsion, hemorrhage, necrosis, inflammatory mass, or cecal neoplasm, right hemicolectomy is advised, though it carries higher morbidity and mortality [4]. Some advantages of suture repair and omentoplasty are shorter hospital stay, less blood loss, easier hemostasis control, and lower risk of anastomosis breakdown [4]. The vital status of the patient is an important factor in deciding on a more invasive procedure [11]. Mortality rates associated with cecal perforation range from 30% to 72% [12]. The outcome of spontaneous colonic perforations depends on the time of onset, degree of peritoneal contamination, and prompt surgical intervention [1].

## Conclusions

This case report describes a rare spontaneous cecal perforation in a 40-year-old pregnant woman who, unlike other cases, displayed none of the probable etiologic factors reported in the literature. The well-being of mother and fetus were at stake, thus prompting appropriate decision-making and subsequent treatment. The perforation was repaired by excision of the edges, primary suture, and omentoplasty. Few recent studies comparing this approach with right hemicolectomy have been done. A larger prospective study is therefore recommended.
